# Editorial: Neuroprotective mechanisms by phytochemicals in neurological disorders

**DOI:** 10.3389/fnins.2023.1149639

**Published:** 2023-03-16

**Authors:** Gaurav Kumar

**Affiliations:** Division of Clinical Research, Department of Biosciences, School of Basic and Applied Sciences, Galgotias University, Greater Noida, Uttar Pradesh, India

**Keywords:** Neurodegenerative disorders (ND), Alzheimer's disease (AD), Parkinson's disease (PD), Phytochemicals, Mesenchymal Stem Cells (MSC)

Neurodegenerative disorders are a complex and diverse group of diseases that affect the nervous system. They are the leading cause of death and disability around the world and are getting more attention as laboratory research is translated into clinical practice (Hussain et al., 2018). These conditions are characterized by the structural and functional dysfunction of neurons in the brain. It has been demonstrated that many plant components, such as phenolic compounds, flavonoids and non-flavonoids, and carotenoids, have neuroprotective effects, that not only slow the advancement of Neurological Diseases but also have preventative capabilities (Naoi et al., 2019). This is because these phytopharmaceuticals have a broad spectrum of action, blocking multiple disease-causing or disease-progressing pathways. In this Research Topic, researchers summarized their state-of-the-art approaches and discoveries on Phytochemicals' neuroprotective mechanisms against Neurological Diseases.

The pathogenesis of Parkinson's disease (PD) and Alzheimer's disease (AD) is significantly influenced by transcription factors, viz. nuclear factor κB (NF-κB), Nuclear factor-erythroid factor 2 (Nrf 2), activator protein-1 (AP-1), Transcription factor EB (TFEB), Specificity Protein-1 (Sp1), and Peroxisome proliferator-activated receptor γ (PPARγ). Chemical compounds isolated from natural sources that target TFEB may make important contributions to the treatment of AD and PD (Rai et al.). In addition, a number of studies have already demonstrated that oxidative stress and mitochondrial dysfunction are important contributors to the pathophysiology and development of PD (Ramya et al.). With the advent of contemporary technologies, phytochemical research has made enormous strides forward. The structural properties and multi-target effects of medicinal plant extracts have piqued the curiosity of researchers. It has been discovered that triptolide and celastrol, the two primary bioactive components of *Tripterygium wilfordii* Hook. F. (TWHF), have anti-inflammatory, immunosuppressive, and anti-tumor effects, and that they can be used in the treatment of neurodegenerative diseases, brain and spinal cord injuries, and epilepsy (Cui et al.). A variety of natural treatments, including those made from medicinal herbs, fruits, and vegetables, are available for the treatment of PD. These naturally occurring chemicals suppress iron accumulation, protein misfolding, proteasomal breakdown, mitochondrial homeostasis, and other neuroprotective activities in addition to their anti-oxidative and anti-inflammatory effects (Rahman et al.).

In recent years, network pharmacology has been utilized in the modern study of Herbal Medicine, since it not only integrates biological data and systematic medicine, but also conforms to the holistic theory, multicomponents, and multitargets of Herbal Medicine. Network pharmacology has the ability to elucidate the mechanisms of complex herbal formulations by identifying bioactive substances and indicators. The application of a network pharmacology approach can be quite useful in the search for phytochemicals with therapeutic benefits against tau pathogenesis in AD. This approach is helpful in determining key tau pathogenesis-related targets like PPARG, PTGS2, CTNNB1, STAT3, IL1B, ESR1, VEGFA, BCL2L1, JUN, and APP (Zeng et al.). This approach can also support the putative mechanism by which phytochemicals and secondary metabolites have substantial potential for reducing CNS viral infection. An ascending viral infection that traveled retrogradely along nerve fibers could cause respiratory failure by affecting the midbrain. The use of phytochemicals with a favorable CNS penetration profile may provide a unique therapeutic option for treating neurotropic viral infections that have become resistant to standard treatment methods (Bhattacharjee et al.).

MSCs are a treatment alternative that can be utilized to investigate diseases, reconstruct models of neurodegenerative disorders, and for cell therapy. MSCs are pluripotent stem cells with the ability to self-renew and differentiate in several directions. MSCs may serve as a dependable source of brain cells for future cell replacement therapies and regenerative medicine treatments (Yao et al., 2020). Most recently, it was found that human mesenchymal stem cells (hMSCs) treated with resveratrol and coenzyme Q10 showed an increase in neural stem cell markers after being damaged by 1-methyl-4-phenylpyridinium (MPP+), a common neurotoxin (Hernández-Pérez et al.). As MSCs and antioxidants have various beneficial effects, they may be a useful tool in the fight against neurodegenerative disorders.

Overall, a series of articles within the current Research Topic have revealed a number of intriguing results that facilitate the comprehension of neurodegenerative disease progression and neuroprotective processes. It can be stated that neurodegenerative illnesses do have potential treatments, however these treatments are not optimal due to their downsides. Several traditional medicinal herbs and their plethora of bioactive phytochemicals need further investigation in order to generate new safe and effective neuroprotective pharmacological agents. However, the usage of phytopharmaceuticals is restricted, and this corresponds to their bioavailability, a vital feature of pharmacokinetics (Sharifi-Rad et al., 2022). From a prospective viewpoint, overcoming the limited bioavailability of phytochemicals is critical, and biotechnological methods will be required, as well as new clinical research employing phytochemicals and derivatives to treat Neurological Diseases (Liu et al., 2022). The investigation of new phytopharmaceuticals for the prevention or treatment of neurodegenerative illnesses is a significant issue for medical research, which must be encouraged to improve the health and quality of life of neurodegenerative disease patients.

We anticipate that this topic will broaden our understanding of phytochemicals' neuroprotective mechanisms in modulating the molecular mediators of neurodegenerative disorders, as well as new challenges and future perspectives in neurodegeneration that will provide exciting insights into new therapeutic approaches to various neurological disorders.

## Author contributions

The author confirms being the sole contributor of this work and has approved it for publication.
